# Large Language Model Framework for Structured Biomarker Extraction and Triple-Negative Breast Cancer Identification From Pathology Reports: Development and Evaluation Study

**DOI:** 10.2196/97117

**Published:** 2026-10-23

**Authors:** Nirmit Jain, Trisha Annambhotla, Akshara Siddharthan, Harshini Kanala, Armaan Budhiraja, Manglam Goutam Sinha, Devyani Charan, Muzaffar Ahmad Dar, Trupti Patel, Biji C L

**Affiliations:** 1 School of Computer Science and Engineering, Vellore Institute of Technology, Vellore Campus, Tiruvalam Road, Katpadi, Vellore, Tamil Nadu, 632014, India, 91 9446171877; 2 School of Bio Sciences and Technology, Vellore Institute of Technology, Vellore, Tamil Nadu, India; 3 School of Advanced Sciences, Vellore Institute of Technology, Vellore, Tamil Nadu, India

**Keywords:** large language model, breast cancer, histology, chain of thought, generative pretrained transformer

## Abstract

**Background:**

Triple-negative breast cancer (TNBC) is an aggressive molecular subtype of breast cancer that needs to be identified accurately from pathology reports to inform decision-making. Large language models (LLMs) have been reported to show great potential in the automatic extraction of important oncology information from unstructured medical text.

**Objective:**

We aimed to create and test an LLM-based approach for automatic biomarker extraction and TNBC subtype classification based on breast cancer pathology reports using prompt-engineering techniques.

**Methods:**

In total, 532 breast cancer pathology reports from The Cancer Genome Atlas (TCGA) were used. Our approach extracted structured oncology features, such as estrogen receptor status, progesterone receptor status, HER2 status, tumor size, tumor grade, and tumor histology, which were used to identify TNBC. We tested 4 frontier models: GPT-4o mini (released July 18, 2024), GPT-4.1 nano (released April 14, 2025), GPT-5 nano (released August 7, 2025), and Gemini 2.5 Flash-Lite.

**Results:**

The designed framework was able to extract relevant biomarkers and clinicopathological attributes from unstructured pathology reports and allowed for the automated identification of patients with TNBC. Gemini 2.5 Flash-Lite showed superior performance in the extraction of clinicopathological attributes. For all models investigated, chain-of-thought (CoT) prompting was shown to outperform other strategies, such as zero-shot and few-shot prompting, for both biomarker extraction and TNBC classification. GPT-4o mini using CoT prompts reported the highest performance for TNBC classification.

**Conclusions:**

Using prompt-engineering techniques based on LLMs proved to be an efficient method of translating unstructured breast cancer pathology reports into structured clinical information. Moreover, it can be used to accurately identify the TNBC subtype.

## Introduction

Breast cancer is one of the major health burdens for women globally, with one of the highest mortality rates. Based on the expression of molecular markers such as estrogen receptor (ER), progesterone receptor (PR), and human epidermal growth factor receptor 2 (HER2), breast cancer is classified into 4 molecular subtypes: Luminal A, Luminal B, HER2-enriched, and triple-negative. In Luminal A breast cancers, ER and/or PR are expressed, but HER2 and Ki-67 are not; Ki-67 is a proliferative marker that shows very little expression. Such tumors are low grade, slow growing, and endocrine-therapy sensitive. In contrast, in Luminal B tumors, ER and/or PR are expressed and either HER2+ or Ki-67 is also present abundantly; Luminal B tumors are of a higher grade than Luminal A tumors and can grow at a faster pace. In the HER2-enriched subtype, HER2 is overexpressed, while ER and PR are absent. Such tumors are highly aggressive but respond well to HER2-targeted therapies. In contrast, triple-negative breast cancer (TNBC) is marked by the absence of these molecular markers: ER, PR, and HER2 are absent or expressed negligibly [1]. It is essential to identify the subtype accurately for precision oncology, as most treatments are strategized based on hormone receptor and HER2 status.

Among all the subtypes of breast cancer, TNBC is a higher-grade cancer with the highest recurrence rates. TNBC is a difficult and complex disease entity for researchers, physicians, and patients, as it is often highly sensitive to chemotherapy in the initial stages but more prone to resistance and relapse [2,3]. In clinical practice, cancers are categorized first by the status of receptors and then refined by biological variables, such as molecular features, histology, and genomic signatures [4,5]. This unique receptor-negative profile not only excludes endocrine and HER2-targeted therapies but also indicates that patients with TNBC have fast disease progression. Therefore, the overall survival outcome remains poorer among patients with TNBC than among patients with receptor-positive cancers [6].

Pathology reports contain information about diagnostic and molecular classifications. These include the presence or absence of biomarkers, such as ER, PR, and HER2, as well as Ki-67 status, along with other histopathological details. Most of this information is unstructured, appearing in the form of free text, which varies in reporting style and has no standardization. With the increasing number of cases of breast cancer, there has been a significant increase in the volume and complexity of pathology reports. This can contribute to an increase in the burden of interpreting these reports and can also be time-consuming. Often, early detection of this type of cancer can save patients’ lives [7]. Conventional methods to study clinical tests computationally involve rule-based natural language processing systems or traditional machine learning techniques. Such methods show practical value, yet they restrict users because they require manual feature development and use fixed rules and structured data. With advances in technology, large language models (LLMs) are now being used to comprehend medical texts [8-10]. However, their accuracy in identifying pathological features has not been sufficiently evaluated. This research gap may limit the adoption of LLM-based approaches in the medical field.

There are multiple models and options that can be used for feature extraction. This paper explores commonly used LLMs paired with suitable prompting techniques and aims to identify the models’ accuracy in extracting biomarkers that are used to determine the status of TNBC [8]. It aims to identify a suitable model that is cost-efficient, fast at processing, and high in accuracy. Accuracy is defined here as the similarity between the LLM’s extracted feature data and manually extracted ground-truth values [11,12]. Additionally, we evaluate different prompting techniques to identify approaches that improve efficiency in downstream clinical data processing. The major contributions of this paper are as follows: (1) development of an LLM pipeline to extract structured oncological features from unstructured pathology reports; (2) evaluation and comparison of multiple prompting strategies, including zero-shot, few-shot, and chain of thought (CoT), on pathology report extraction tasks; and (3) development and evaluation of an automated approach for identifying TNBC cases from unstructured pathology reports using LLM-based information extraction.

## Methods

### Background

We created an LLM framework to extract oncological biomarker features and identify TNBC subtype statuses from pathology reports, as depicted in Figure 1.

**Figure 1.**
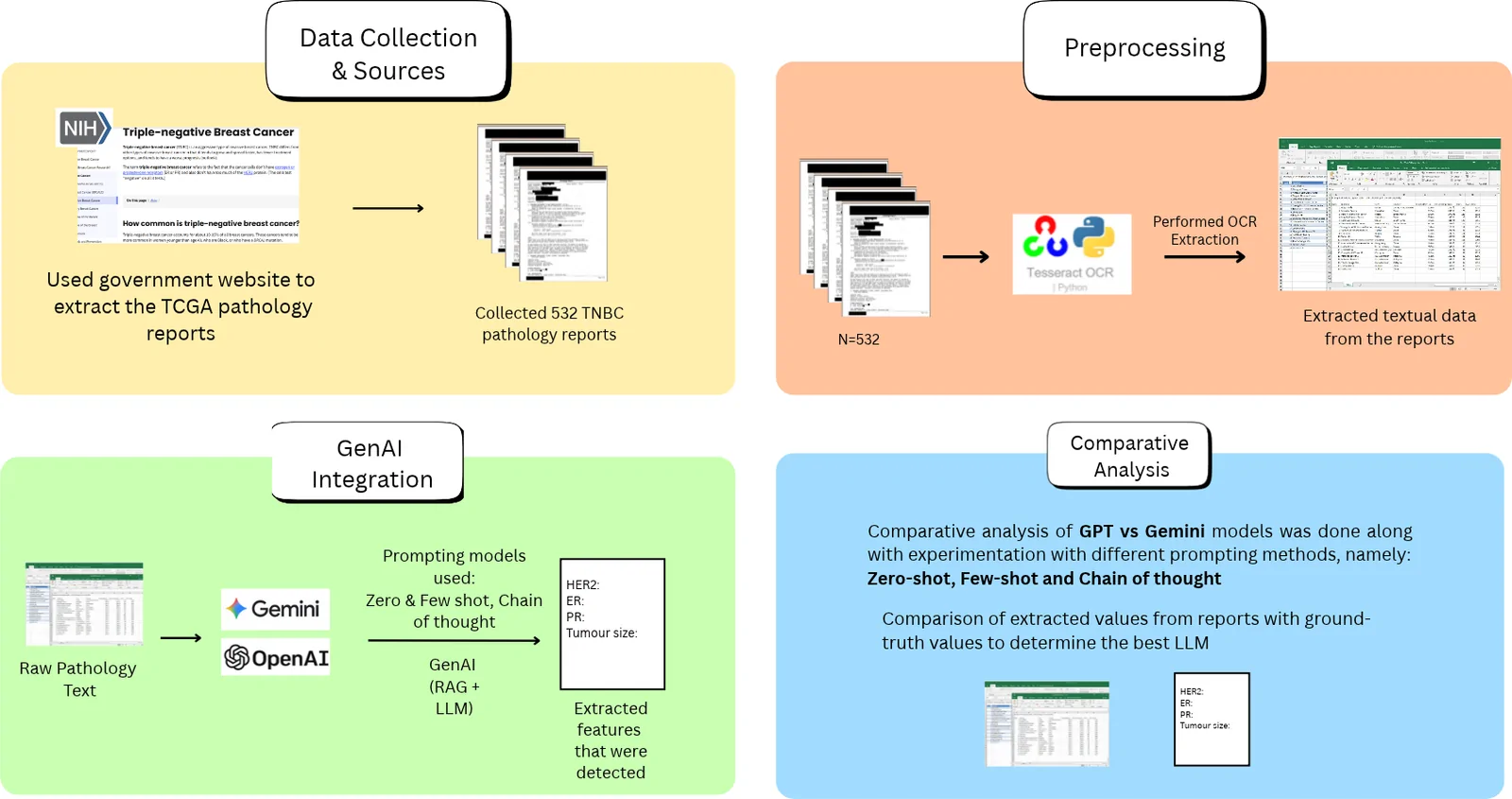
Suggested process flow for triple-negative breast cancer pathology report analysis: data gathering, optical character recognition (OCR) preprocessing, generative AI (genAI) biomarker extraction (GPT vs Gemini), and comparative dashboard creation. LLM: large language model; RAG: retrieval-augmented generation; TCGA: The Cancer Genome Atlas.

### Data Collection and Sources

The dataset used in this analysis was obtained from The Cancer Genome Atlas/Genomic Data Commons (TCGA/GDC) data portal. The reports were downloaded as PDF files using the GDC API. Each downloaded pathology report was assigned the corresponding GDC file’s universally unique identifier (UUID) to ensure traceability throughout preprocessing, feature extraction, and evaluation. A total of 532 pathology reports, including both scanned and text-based documents, were included in this study. Feature selection was based on a curated Breast Cancer Feature Checklist consisting of 4 clinically relevant domains, as shown in Figure 2. First, core receptor status, such as ER, PR, HER2, and TNBC definition, was selected, which helped differentiate hormone receptor–positive, HER2-enriched, and triple-negative breast cancer. Receptor status helps in choosing systemic therapy. Second, tumor status, such as tumor size category, histologic grade, and histology type, was selected, as this reflects primary tumor burden and intrinsic aggressiveness. Third, proliferation and molecular markers, such as Ki-67 index, p53 status, and the basal markers CK5/6 and EGFR, were selected because they are associated with tumor growth rate. Finally, nodal status, such as lymph node stage and lymphovascular invasion, was selected because it reflects metastatic potential. However, since the pathology reports were not uniform, not all features were obtained from all reports.

**Figure 2.**
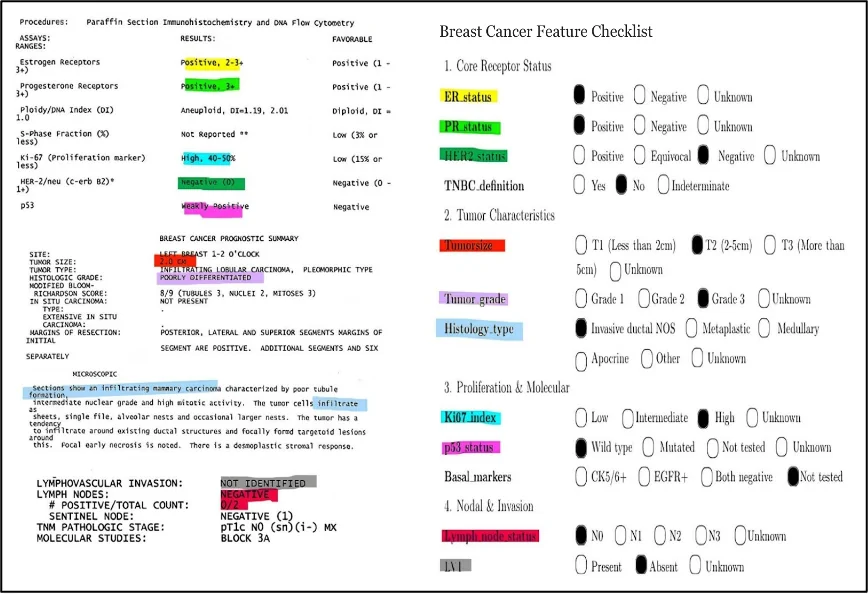
Extraction of pathology features related to oncology based on large language model framework: manually annotated pathology report of triple-negative breast cancer (TNBC; left) mapped against the corresponding biomarker checklist (right).

### Preprocessing

#### Overview

Along with their lack of uniformity, pathology reports tend to exceed optimal context size. Also, there are sections in pathology reports that are interrelated. To maintain semantic integrity, a hierarchical chunking approach was used. Information dilution and loss are common problems when working with long clinical documents such as pathology reports, and the semantic chunking approach is a common solution [13]. Using a sliding window approach, the pathology reports were segmented based on clinically relevant boundaries, such as immunohistochemistry, diagnosis, and microscopic findings. We used optical character recognition (OCR) technology, which is used for unstructured data formats such as scanned documents. PDFs of the pathology reports in our dataset were first converted to images with a resolution of 300 DPI through the pdf2image package. The OCR process was then carried out with the use of the pytesseract tool in Tesseract OCR (version 4.x), which processed each page independently. The resulting page-by-page texts were combined using pytesseract to form the whole pathology report before any further processing. This technology was necessary, as a real-world scenario may include handwritten notes, unclear scanned documents, or documents in which pages have been rotated. We achieved a high volume of report processing with OCR technology by using parallelization through Python’s ProcessPoolExecutor package. Each pathology report was considered a different task to be processed by a dedicated worker, which improved the speed of preprocessing without changing OCR parameters. Furthermore, each report was treated as a separate task that had to be executed by a worker process using multiple central processing unit threads, which significantly accelerated the process. The extracted text chunks were input into the LLM using schema-constrained prompts that ensured structured output in JSON format with fields for ER_status, PR_status, HER2_status, and Tumor_size [14]. For few-shot and CoT prompting methods, more biomarkers, namely, Ki-67 and p53, were also extracted if present in the pathology report. Missing biomarkers were clearly noted as “not reported” rather than guessed by the model. For the categorical biomarkers (ER, PR, HER2), accuracy was computed as exact label agreement between the LLM-extracted value and the manually curated ground truth. For tumor size, which is reported in free text with heterogeneous units and varying dimensions, the largest reported dimension was first extracted programmatically (with mm to cm normalization applied where appropriate) and then mapped to one of three clinically relevant size categories (<2 cm, 2‐5 cm, >5 cm). Tumor size accuracy therefore reflects category-level agreement between the predicted and ground-truth size bins rather than exact numerical or tolerance-based agreement on the raw measurement.

#### Generative AI Integration

Pretrained LLMs from Google’s Gemini family and OpenAI’s GPT series were evaluated for structured extraction from pathology text [15-17]. To maximize structured data extraction from clinical texts, 4 prompting methods were studied: zero-shot, one-shot, few-shot, and CoT. In all methods, the same set of pathology reports was used and only the prompts were varied, in order to observe the impact of prompt engineering. This produced accurate identification of target features and their corresponding values. Categorizing breast tumors based on receptor status helps to predict outcomes and assist in the management of breast cancer [18]. A lightweight knowledge base was constructed based on World Health Organization immunohistochemical (IHC) interpretation rules. To reduce variation due to terminological inconsistency, synonym normalization was included in the prompt specifications. Thus, terms such as “estrogen receptor” and “ER receptor” were normalized to the term “ER,” while “HER2,” “HER-2,” “HER2/neu,” and “HER2 amplification” were normalized to the term “HER2.” The rules stated that both ER and PR are negative if their reported value is less than 1% and positive if it is greater than or equal to 1%. HER2 was interpreted on the basis of the IHC score. In this scoring system, a score of 0 or 1+ is interpreted as negative, a score of 2+ it is interpreted as equivocal or borderline, and a score of 3+ is interpreted as positive. Our knowledge base was injected during prompting conditioning and was also used in OCR-extracted data, which were aligned to the curated biomedical markers. This helped in reducing latency and avoiding OCR-driven retrieval failures, which were observed in early experiments.

Our goal was to find the LLM that provided the best possible results while performing feature extraction. We examined 3 OpenAI models (GPT-4o mini, released July 18, 2024; GPT-4.1 nano, released April 14, 2025; and GPT-5 nano, released August 7, 2025) and 1 Google model (Gemini 2.5 Flash-Lite). GPT-4o mini was integrated using API calls. It used the instruction hierarchy method, which improved security and also increased robustness by almost 63%. This model is also more economical in comparison [19]. In terms of following instructions, GPT-4.1 nano outperformed GPT-4o mini. GPT-4.1 nano has a better context window and improved long-context comprehension, a benchmark used to evaluate an LLM’s instruction-following ability. This nano model is the fastest and least expensive of the GPT models we tested. It has a context window of 1 million tokens and scored 80.1% on the Massive Multitask Language Understanding (MMLU) benchmark. Gemini 2.5 Flash-Lite is fast and cost-efficient, works well with high volumes, and is latency sensitive for classification-related tasks. Gemini 2.5 models have shown improvements in coding tasks, mathematics, reasoning, and image understanding. The model we tested has a context window of 1 million tokens and an MMLU benchmark score of 72.9%.

The OpenAI language models were queried through the Chat Completions API, while Gemini 2.5 Flash-Lite was tested via the google.generativeai.GenerativeModel framework. In all experiments, the temperature value was set to 0 to generate deterministic results.

#### Prompting Strategies

We used 3 prompting strategies. The zero-shot strategy is used to generate a response based on its pretrained knowledge and generalization ability without any guidance on format, style, or reasoning. The prompt provides only a question or instruction; therefore, the model interprets the task on its own, which makes this strategy flexible but sometimes less predictable. It is preferred when minimal prompting is desired or examples are unavailable [20].

A few-shot prompt includes a few examples to guide the model to respond in a particular way. The included reasoning style, output format, and level of detail lead to more controlled and reliable responses. This strategy is useful for complex tasks or when consistent output quality is required.

CoT prompting takes the form of logical steps and can deliver accurate results with reduced errors. In this strategy, no examples are provided to the LLM; instead, only the methodology for arriving at a logical way to process the instructions is injected [21].

For the one-shot prompting approach, the system was initially presented with an example pathology report along with structured JSON output. In few-shot prompting, in addition to the example pathology report and JSON output, examples of several other annotated pathology reports demonstrating the extraction of tumor size, ER/PR status, HER2 score, Ki-67 category, and the structure of the output were also shown to the model. In CoT prompting, the model was asked to extract step-wise evidence followed by generating the structured output.

The main objective of this task was to mine the clinicopathological biomarkers as structured data from the unstructured pathology reports. The main variables mined were tumor size, ER status, PR status, and HER2 status. In few-shot and CoT prompting, Ki-67 and p53 information was also mined wherever available. The clinical rules were encoded in the prompts, such as ER/PR positivity (greater than or equal to 1%), HER2 score interpretation (0‐1+, 2+, 3+), and Ki-67 categories (<10%, 10%‐20%, >20%).

Gemini 2.5 Flash-Lite and the 3 GPT models were considered in order to observe variance in model robustness. Various prompting techniques, as shown in Figure 3, were studied to improve the overall performance of the LLM models. The overall accuracy performance was calculated as an average of the accuracies per pathological feature, namely, HER2, ER, PR, and tumor size, for each pairing [22,23].

**Figure 3.**
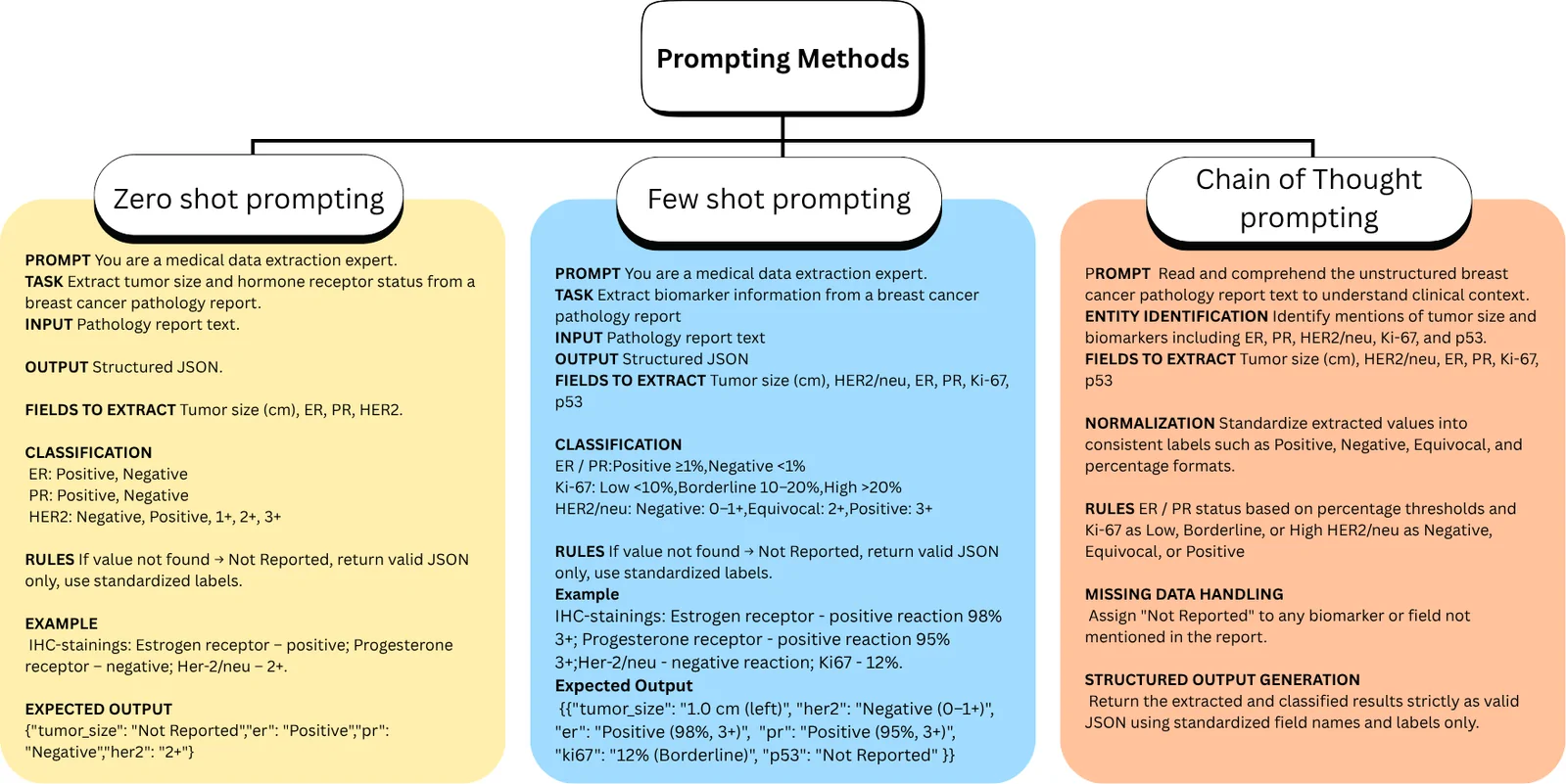
Prompt-engineering techniques that are structured to facilitate the extraction and standardization of clinically important breast cancer biomarkers: tumor size, estrogen receptor (ER), progesterone receptor (PR), human epidermal growth factor receptor 2 (HER2), Ki-67, and P53).

Automated extraction was performed using the json.loads() function in Python for model responses. In cases where parsing was not successful, the extraction process took the first JSON object inside the braces and repeated the parsing process. A default value of “not reported” was used to fill in missing fields. API errors were handled via try-except statements, with unsuccessful API calls resulting in an error label for all extraction fields. Reports that had insufficient OCR text were skipped and labeled as such. Extracted biomarkers were then exported to Microsoft Excel via the openpyxl package. The automated extraction pipeline incorporated comprehensive error-handling methods such as try-except statements to catch failures during API calls and heuristics to exclude reports with low amounts of text extracted by OCR. Although these error-handling mechanisms proved invaluable for achieving robustness of the extraction process, no reports were filtered out from the analysis because of fatal API errors. Thus, 532 pathology reports were evaluated with each model and prompting strategy. If the report contained only a partial parsing error or if a model failed to extract one of the biomarkers, then the corresponding field value was set to “not reported.” Importantly, the values were not filtered out from the dataset and were scored using ground-truth labels. Therefore, a failure in extraction resulting in a “not reported” label of a present biomarker would be counted as a false negative.

#### Performance Metrics and Statistical Analysis

To standardize the performance measures of each model in extracting a biomarker, we used a 4-tier taxonomy: positive, negative, equivocal, and not reported (Table 1). Confusion matrices for all 4 features using all combinations of models and prompting strategies were computed to compare performances. Evaluation of the models’ performance was conducted based on the following metrics: accuracy, precision, recall, *F*_1_-score, and specificity.

**Table 1.** Standardized taxonomy for the evaluation of oncology feature extraction.

Category	Matched from
Positive	Contains “positive” or is “3+” / “3”
Negative	Contains “negative” or is “0” / “1+” / “1”
Equivocal	Contains “equivocal” or is “2+” / “2”
Not reported	Blank/NaN^a^ or contains “not,” “pending,” “skipped”

a NaN indicates instances when the data-extraction algorithm encountered a medical record where the test result field was completely empty, unrecorded, or missing.

For robust statistical evaluation and to compute reliable CIs for the prediction accuracy of each model–prompting strategy pairing, we used a nonparametric bootstrap algorithm with replacement. For all combinations of model, prompting strategy, and biomarker feature (HER2, ER, and PR) separately, the bootstrap process was performed 2000 times. The individual pathology report was taken as the unit of sampling to account for the underlying dependency structure of the data, such that predictions derived from the same pathology report would not be sampled independently of each other. A resampled index set identical to the original matched dataset in size was created, and the overall accuracy statistic was recomputed solely on the bootstrap sample in each iteration. The 95% CIs were computed using the percentile bootstrap approach, which is based on the 2.5th and 97.5th percentiles of the 2000 resampled accuracy distributions. While bootstrapping was performed independently for each model-prompt-feature combination due to the design of our experiment, in that each combination was trained separately on its own matched dataset, no further stratification by either true class label or pathology report covariates was applied. With regard to handling missing or incorrect inferences and predictions, those that were marked with “skipped” or “error” labels were not dropped from the study but instead normalized to the “not reported” class during preprocessing. They were then incorporated into the computation of accuracy as well as the bootstrapping process. This conservative method guaranteed that the computed CIs represent the true failure rate of the models and do not artificially inflate model performance through omission of unprocessed instances. Finally, in order to ensure the full reproducibility of the results, a random seed of 42 was used for all resampling processes.

#### TNBC Classification and Evaluation

We report the evaluation of TNBC classification using accuracy, precision, recall, specificity, and *F*_1_-score. The measures were computed using the categories of true positive, true negative, false positive, and false negative based on the available ground-truth labels.


$$\text{Accuracy=}\frac{\text{TP+TN}}{\text{TP+TN+FP+TN}}$$

$$\text{Precision=}\frac{\text{TP}}{\text{TP+FP}}$$

$$\text{Recall=}\frac{\text{TP}}{\text{TP+FN}}$$

$$\text{Specificity=}\frac{\text{TN}}{\text{TN+FP}}$$

$$F1=2\times \frac{\text{Precision}\times \text{Recall}}{\text{Precision}+\text{Recall}}$$

where TP means true positive, TN means true negative, FP means false positive, and FN means false negative.

### Ethical Considerations

Deidentified pathology reports of patients with breast cancer were sourced from The Cancer Genome Atlas (TCGA) via the Genomic Data Commons (GDC) Data Portal. Since the experiment only analyzed secondary, deidentified data, no need for ethics committee approval and informed consent was required due to the nature of the experiment itself. All data were accessed and analyzed according to the rules set forth by TCGA/GDC. In future deployment in clinics, proper patient data privacy should be observed when using commercial LLMs.

Although this study evaluated commercial large language models through API-based inference, only deidentified pathology reports from The Cancer Genome Atlas were processed. In real-world clinical deployment, identifiable pathology reports should undergo appropriate deidentification before external processing. Institutions should ensure compliance with applicable privacy regulations and evaluate the security implications of transmitting clinical data to third-party AI services.

## Results

Four different LLMs were compared based on 3 prompting methods (zero-shot, few-shot, and CoT) for the extraction of 4 clinical features (HER2, ER, PR, and tumor size) from pathology reports. Great variability in performance was seen among models, prompting methods, and targeted clinical features. As reported in Table 2, out of all of the models analyzed, Gemini 2.5 Flash-Lite performed the best, with an accuracy of 91.38%, *F*_1_-score of 86.48%, and precision of 87.81%. GPT-5 nano produced the highest accuracy (91.69%), but its precision and recall values were slightly lower than Gemini’s. The highest specificity value (93.39%) was observed for GPT-4o mini, which means this model showed the best performance in recognizing negative samples; however, its precision (78.39%) and recall (77.48%) values were relatively low. GPT-4.1 nano showed the lowest accuracy value (87.61%), but its precision-recall balance was moderate (*F*_1_-score=81.46%).

**Table 2.** Performance of large language models in extracting oncology features.

Model	Accuracy, %	Precision, %	Recall, %	*F*_1_-score, %	Specificity, %
GPT-4o mini	89.22	78.39	77.48	77.65	93.39
GPT-4.1 nano	87.61	84.70	80.17	81.46	88.14
GPT-5 nano	91.69	87.32	84.73	85.76	91.24
Gemini 2.5 Flash-Lite	91.38	87.81	85.63	86.48	92.17

The Gemini 2.5 Flash-Lite model is known for its feasibility in workflows consisting of bulk data, meeting the requirements to perform sensitive tasks (eg, extraction tasks for which errors carry substantial clinical consequences, such as determining ER, PR, and HER2 receptor status, which directly informs the classification of TNBC). This model shows strong dependence on structured prompting. CoT prompting was superior to both zero-shot and few-shot prompting in terms of the extraction of molecular markers (ER, PR, and HER2). For instance, in the Gemini 2.5 Flash-Lite model with CoT prompting, the highest *F*_1_-score was observed for the HER2 marker (85.61%), which was far better than with zero-shot (54.32%) and few-shot (43.56%) prompting. On the other hand, there was no clear advantage shown for few-shot prompting compared to zero-shot prompting for some models, including GPT-4.1 nano, for which zero-shot and few-shot showed exactly the same performance for all targets.

The key takeaway from our analysis is the trade-off between precision and recall and between accuracy and specificity among the models and prompting approaches. Models with high precision often had low recall and vice versa. For instance, GPT-5 nano with zero-shot had very good precision for tumor size (68.96%) but low recall (51.30%), meaning that even though the model was accurate in its prediction, it often failed to detect true positives. On the other hand, Gemini 2.5 Flash-Lite with zero-shot had better recall (75.79%) compared to precision (74.73%). GPT-4o mini was the model with the highest specificity (up to 96.76% for PR) but the lowest accuracy in terms of positive predictions (precision). It seems that the model is very conservative in its prediction, meaning that it tries not to generate false positives. On the other hand, GPT-5 nano with zero-shot generated predictions with much lower specificity (77.78% for HER2, 81.55% for ER). The task of HER2 status extraction showed the highest variability and depended greatly on both the model used and the prompting approach applied. The best *F*_1_-score and precision were observed for Gemini 2.5 Flash-Lite with CoT (*F*_1_-score=85.61%, precision=91.14%), whereas GPT-4o mini with few-shot prompting showed the worst results (*F*_1_-score=50.92%). It is important to mention that zero-shot prompting produced superior results compared to few-shot prompting for HER2 status extraction in all tested models except for GPT-4o mini, where the best *F*_1_-score was obtained using CoT (56.58%). This task appears to be complicated due to various conventions of reporting HER2 status. The extraction of tumor size information was a much tougher task than others, with varying results depending on the model used. The best result was recorded by GPT-5 nano with CoT (*F*_1_-score=76.28%, accuracy=83.37%), while the poorest result was achieved through the use of GPT-4o mini with CoT (*F*_1_-score=68.49%, accuracy=73.16%). The poor results for tumor size information might be attributed to the differences in reporting format (such as 2.3 cm, 23 mm, and pT1c), among other reasons.

We calculated 95% CIs for accuracy using bootstrapping for each model, strategy, and target (HER2, ER, PR), as reported in Table 3. The results showed that consistent improvement was made possible with CoT prompting with higher point estimates and narrower CIs in comparison to zero-shot and few-shot strategies. For example, GPT-4o mini reached an accuracy of 0.938 to 0.973 when HER2 was used as the target, a result considerably better than in zero-shot and few-shot cases, with an accuracy interval of 0.765 to 0.833. This improvement can be seen for both ER and PR targets, with a CI width around 0.035 to 0.042. Also, there were clear differences for each model. GPT-4.1 nano had a rather low level of accuracy in zero-shot and few-shot conditions (HER2, 0.721‐0.794); nevertheless, with CoT prompting, the accuracy interval became much higher (0.922‐0.962). In the case of GPT-5 nano, the pattern is somewhat opposite: although the model showed a rather poor level of zero-shot accuracy (intervals centering at 0.65‐0.73 for ER and PR), CoT prompting increased the accuracy above 0.93 with narrow CIs. Finally, Gemini 2.5 Flash-Lite with CoT prompting had the smallest CI width in PR (0.038), ER (0.036), and HER2 (0.033). CI widths helped to prove the reliability of CoT prompting for the biomarker extraction task.

**Table 3.** Bootstrap 95% CIs by model, prompting strategy, and extraction target (human epidermal growth factor receptor 2 [HER2], estrogen receptor [ER], and progesterone receptor [PR]), with 95% CI widths indicating stability of estimates.

Model and prompting strategy	Feature	Accuracy 95% CI	CI width
GPT-4o mini			
Zero-shot	HER2	[0.765, 0.833]	0.068
Zero-shot	ER	[0.921, 0.961]	0.04
Zero-shot	PR	[0.914, 0.956]	0.042
Few-shot	HER2	[0.765, 0.833]	0.068
Few-shot	ER	[0.912, 0.954]	0.042
Few-shot	PR	[0.903, 0.948]	0.045
CoT^a^	HER2	[0.938, 0.973]	0.035
CoT	ER	[0.930, 0.968]	0.038
CoT	PR	[0.912, 0.954]	0.042
GPT-4.1 nano			
Zero-shot	HER2	[0.721, 0.794]	0.073
Zero-shot	ER	[0.851, 0.906]	0.055
Zero-shot	PR	[0.851, 0.906]	0.055
Few-shot	HER2	[0.721, 0.794]	0.073
Few-shot	ER	[0.851, 0.906]	0.055
Few-shot	PR	[0.851, 0.906]	0.055
CoT	HER2	[0.922, 0.962]	0.04
CoT	ER	[0.895, 0.941]	0.046
CoT	PR	[0.847, 0.903]	0.056
GPT-5 nano			
Zero-shot	HER2	[0.860, 0.914]	0.054
Zero-shot	ER	[0.656, 0.734]	0.078
Zero-shot	PR	[0.648, 0.727]	0.079
CoT	HER2	[0.930, 0.967]	0.037
CoT	ER	[0.930, 0.967]	0.037
CoT	PR	[0.916, 0.957]	0.041
Gemini 2.5 Flash-Lite			
Zero-shot	HER2	[0.840, 0.898]	0.058
Zero-shot	ER	[0.912, 0.954]	0.042
Zero-shot	PR	[0.904, 0.949]	0.045
Few-shot	HER2	[0.847, 0.903]	0.056
Few-shot	ER	[0.883, 0.932]	0.049
Few-shot	PR	[0.870, 0.922]	0.052
CoT	HER2	[0.942, 0.975]	0.033
CoT	ER	[0.930, 0.968]	0.038
CoT	PR	[0.912, 0.954]	0.042

a CoT: chain of thought.

Out of 53 actual TNBC cases (according to the golden standard of TNBC that involves the ER-, PR-, and HER2-negative status), 45 cases were detected correctly using the most effective pairing, the GPT-4o mini model with CoT prompting, while 8 cases were misclassified as non-TNBC. Out of 479 actual non-TNBC cases, 477 cases were detected correctly, with only 2 false-positive detections. However, the discrimination ability of the TNBC cases is excellent. GPT-4o mini using CoT prompts demonstrated the highest sensitivity of 84.9% for TNBC detection. It had a specificity of 99.6%, which demonstrates perfect classification of non-TNBC cases with an extremely low false-positive rate of 0.4%. The performance of GPT-5 nano using zero-shot prompting was slightly inferior in sensitivity (83.0%) compared to GPT-4o mini using CoT (84.9%) while maintaining perfect specificity (99.6%), which accurately detected 477 out of 479 non-TNBC cases. The minor difference in sensitivity (1.9%) between the two models indicates that both models are practically suitable for TNBC diagnosis. Supplementary figure S12 in Multimedia Appendix 1 shows the confusion matrix for TNBC detection.

## Discussion

### Principal Findings

A critical consideration in deploying LLMs for clinical applications is the risk of hallucination. Getting results with greater accuracy and minimizing hallucinated results may be the scope of future research. Hallucination is a common complication during feature extraction. While direct hallucination quantification was beyond the scope of this study, the observed precision-recall trade-offs provide indirect evidence of extraction reliability. Zero-shot prompting of models with lower precision, such as GPT-4o mini for HER2 extraction (precision, 56.40%‐59.57%), may be at elevated risk of false-positive extractions. Conversely, models with lower recall, such as GPT-5 nano with zero-shot prompting for ER extraction (recall, 62.20%), may be more likely to miss true values than to fabricate them. These performance characteristics should inform model selection based on the clinical priority of minimizing false positives versus false negatives. Due to various factors determining tumor size, as well as indirect references to immunohistochemistry, it is essential to extract contextually and factually accurate information [24,25]. Therefore, a thorough ground-truth analysis was performed manually with domain experts to check the precision of the model extraction. For instance, various sizes of tumor were present in most of the pathology reports, and thus, determining the largest circumference and most detrimental carcinoma was extremely crucial. Prompt generation must be specific and succinct. CoT prompting applies this technique of performing a deeper analysis of the meaning of the IHC indicators and extracting the relevant values. Tumor size had different formats throughout the pathology reports, and hence, extracting not only the largest diameter but also the complete dimensions became necessary. This had a better outcome in few-shot prompting through the use of examples given on how to extract the dimensions [26,27]. Thus, prompt generation requires being extremely specific and providing a clear context for medical terms as well as pairing prompts with proper examples to extract information in the desired manner. These strategies help in reducing hallucinations and can also be used for training future models.

In the current study, results show that prompt engineering is crucial in boosting the performance of clinical information extraction from heterogeneous pathology reports. This observation is similar to the conclusions drawn in recent studies, in which reasoning greatly boosts the performance of LLMs in clinical natural language processing due to its ability to facilitate structured reasoning of the narrative [28-31]. The better performance demonstrated by CoT prompting is due to the ability of CoT prompting to help the LLM break down complex extraction tasks into reasoning steps prior to responding. The pathology reports of breast cancer have free-text narratives, inconsistency in style of reporting, multiple lesions, and variations in IHC format. This makes the task more complex, as it needs more than matching with keywords. CoT prompting is helpful for accurately identifying clinical biomarkers without any ambiguity. The advantages of reasoning-based prompts have also been highlighted by several studies related to clinical summarization, information extraction, and question answering in medicine [12,32].

From the analyzed models, Gemini-2.5 Flash-Lite showed the best overall accuracy in extraction despite being one of the smallest models, implying improvement in the domain of instruction following and reasoning skills. Therefore, small, instruction-tuned models are more practical due to their efficiency and better computational capabilities [28,30]. Moreover, the comparison showed that the prompt design impacted the extraction performance of all models, showing that the optimization of prompts is just as crucial as the choice of the model in healthcare tasks.

In line with previous studies, extraction accuracy was higher for ER, PR, and HER2 status compared to tumor size [33]. However, hormone receptor status is usually expressed in a standardized language, while tumor size may involve various measurements, macro- and microdimensions, or even multiple lesions in one report. Therefore, contextual reasoning is needed to recognize the clinically relevant measurement of the primary tumor. This demonstrates that the performance of extraction depends not only on the model but also on the complexity of pathology reports. Reasoning-based models for LLMs are able to provide help to pathologists as they are capable of extracting clinically relevant markers from unstructured pathology reports through automation. However, expert review is necessary prior to clinical application.

Hallucination is an important drawback that hinders the clinical implementation of LLMs. Ambiguous language, multiple numerical measurements, and indirect indication of immunohistochemistry in the pathology reports may lead to clinically plausible but incorrect results. To prevent this problem, manual verification of each extracted variable was conducted in the current study. Prompt engineering cannot solve the issue completely, and human verification is still needed [34,35].

This research faces certain limitations. It used 532 pathology reports and considered only 4 important clinical parameters (ER, PR, HER2, and tumor size). Generalizability needs to be enhanced through validation on more comprehensive datasets at multiple sites and through consideration of other pathological parameters. In addition, using proprietary LLM models may impact reproducibility due to the constant development of these commercial models. Thus, future research should explore medical LLM models for specific domains, as well as RAG methods and clinical validation.

In summary, the results show that reasoning-driven prompt engineering improved the performance of LLMs in identifying clinically important biomarkers from unstructured breast cancer pathology reports. The consistently superior results obtained using the CoT prompting method in various models illustrate the potential of prompt engineering as a viable approach to automating the extraction of clinical data. Innovations in the fields of prompt engineering, prevention of hallucinations, and external validation on different clinical datasets, as well as the creation of medically validated LLMs, will pave the way for the incorporation of AI in digital pathology and precision oncology pipelines [28,29,35].

### Limitations and Future Scope

One limitation of the current study is the absence of baseline evaluation when compared with other approaches for information extraction, which include the rule-based or regular expression–based approaches. Since the aim of the current research was to conduct evaluation of lightweight LLMs with different prompt formats, the comparison with rule-based approaches was out of scope. Future research should conduct analyses of the computational efficiency, implementation ease, and performance of LLM approaches compared with traditional rule-based approaches.

The current study was conducted on 1 dataset only, comprising 532 TCGA breast cancer pathology reports. While the dataset allowed us to perform a comprehensive comparison among different prompting approaches, future research must be conducted on multi-institutional pathology reports written in different styles.

Another limitation is that the current study concentrated on finding clinically relevant biomarkers (ER, PR, HER2, and tumor size) and TNBC. Classification of other molecular subtypes, such as Luminal A, Luminal B, and HER2-enriched breast cancer, and TNM staging based on American Joint Committee on Cancer criteria were out of the scope of the study. The future work would include integration of more pathological factors in order to perform classification of other molecular subtypes and TNM staging automatically.

In the current study, the tested LLMs produced categorical predictions but not probability-based predictions; thus, receiver operating characteristic–area under the curve analysis could not be performed. In future work, confidence-based prompting techniques or probability-based LLM output would need to be considered to perform threshold-based performance analysis.

While GPT-5 nano demonstrated 83% sensitivity for TNBC detection, this false-negative rate is not adequate for autonomous clinical decision-making. Thus, the proposed framework should be considered a clinical decision support framework that requires review by a professional pathologist. Finally, reproducibility is bound to change with future model developments of commercials LLMs. Validation through domain-specific medical LLMs, RAG, and clinical validation will add more robustness to the proposed framework.

This study did not follow a formal AI-diagnostic reporting checklist such as TRIPOD-AI (Transparent Reporting of a Multivariable Prediction Model for Individual Prognosis or Diagnosis Using Artificial Intelligence) or STARD-AI (Standards for Reporting of Diagnostic Accuracy Studies Involving Artificial Intelligence). While model settings and error-handling procedures were reported, dataset flow and prompt templates were described narratively rather than as a fully reproducible pipeline, and ground-truth annotation protocol details (number of annotators, interannotator agreement) along with external validation on independent, multi-institutional datasets were outside the scope of the original study design. Future work will adopt a formal AI-reporting checklist and incorporate multiannotator ground-truth curation with agreement statistics, along with prospective external validation, to strengthen the reproducibility and clinical generalizability of this framework.

### Conclusions

This study highlights the use of LLMs with appropriate prompting techniques for oncology feature extraction from pathology reports. Gemini 2.5 Flash-lite, GPT-5 nano, and GPT-4o mini achieved the highest accuracy in biomarker status extraction. Among these, GPT-5 nano paired with CoT prompting gave the best results in the detection of tumor size. This finding shows that smaller and more cost-efficient nano models can give better results compared to larger models for information extraction in clinical documents when prompted properly. However, GPT-5 nano had considerably higher processing time for all reports. This could make it less suitable for large-scale deployment. It has immense potential in assisting pathologists and oncologists by automating the extraction of key pathological features from unstructured reports, thereby increasing efficiency and reducing the possibility of human error. This would minimize manual efforts and improving clinical workflow efficiency. Furthermore, when integrated with agentic model context protocol–based workflows [36,37], such systems could continuously validate extracted results, flag TNBC cases in real time, and trigger clinical actions, leading to an AI-driven precision oncology system. This could transform how high-risk breast cancer subtypes such as TNBC are identified and managed in routine clinical practice [38,39].

## Supplementary material

10.2196/97117 Multimedia Appendix 1 Additional experimental details on structured prompting approaches for the extraction of oncology features.

## Abbreviations

CoT: chain of thought
ER: estrogen receptor
GDC: Genomic Data Commons
HER2: human epidermal growth factor receptor 2
IHC: immunohistochemical
LLM: large language model
MMLU: Massive Multitask Language Understanding
OCR: optical character recognition
PR: progesterone receptor
TCGA: The Cancer Genome Atlas
TNBC: triple-negative breast cancer
UUID: universally unique identifier
