# Supplementary material for: Large Language Model Framework for Structured Biomarker Extraction and Triple-Negative Breast Cancer Identification From Pathology Reports: Development and Evaluation Study
Source: JMIR Form Res. 2026 Oct 23;10:e97117. doi: 10.2196/97117 (PMC13694010; doi:10.2196/97117)
Supplement: Supplementary file 1 [file formative-v10-e97117-s001.docx]

**Additional Experimental Details on Structured Prompting approaches for the extraction of Oncology features**

We employed different prompting for information extraction from breast cancer pathology reports. In order to ensure consistency in the structured output, we created a prompt template that includes five key elements (1) role assignment, (2) task specification, (3) format constraints, (4) in-context examples and (5) uncertainty handling. The prompt template for different techniques used in the analysis is listed below.

1. **Zero shot prompt:**

You are analyzing a breast cancer pathology report.

Extract the following fields (return valid JSON only):

- tumor_size (in cm)

- er ("Positive" or "Negative")

- pr ("Positive" or "Negative")

- her2 ("Negative", "Positive", "1+", "2+", or "3+")

-ki67 ) (percentage)

- p53 ("Positive", "Negative", or "Not Reported")

If any field is missing, use "Not Reported".

Return only a valid JSON object with keys:

tumor_size, er, pr, her2, ki67,p53.

2. **Few shot prompting prompt:**

You are a medical data extraction system.

Analyze the following breast cancer pathology reports and extract biomarker information.

Extract and return the following fields as a valid JSON object:

- tumor_size (in cm)

- her2 ("Negative", "Equivocal", "Positive", "Ordered, result pending", or "Not Reported")

- er ("Positive", "Negative", or "Not Reported")

- pr ("Positive", "Negative", or "Not Reported")

- ki67 (percentage + category: Low / Borderline / High / Not Reported)

- p53 ("Positive", "Negative", or "Not Reported")

Reference Ranges (for classification only, do not include in output):

- ER/PR: Negative <1%, Positive ≥1%

- Ki-67: Low <10%, Borderline 10–20%, High >20%

- HER2/neu: Negative 0–1+, Equivocal 2+, Positive 3+ (reflexed to FISH if equivocal)

Output Format Example:

{{

"tumor_size": "1.0 cm",

"her2": "Negative (0–1+)",

"er": "Positive (98%, 3+)",

"pr": "Positive (95%, 3+)",

"ki67": "12% (Borderline)",

"p53": "Not Reported"

}}

If a field is not found in the report, explicitly return "Not Reported".

**3. Chain of thought prompt**

You are a medical data extraction system. Do NOT reveal internal chain-of-thought or private model reasoning.

Instead, produce a concise, external step-by-step extraction log (1-3 short bullets per biomarker) called "extraction_rationale".

Task:

From the pathology report below, extract and return a JSON object with these keys:

- tumor_size (string; include units and laterality if present, or "Not Reported")

- her2 (one of: "Negative", "Equivocal", "Positive", "Ordered, result pending", or "Not Reported")

- er (one of: "Positive", "Negative", or "Not Reported"; if percent/score present include, e.g. "Positive (98%, 3+)")

- pr (same format as ER)

- ki67 (percentage + category: "12% (Borderline)" or "Not Reported")

- p53 (one of: "Positive", "Negative", "Not Reported")

Also include:

- extraction_rationale: a JSON object mapping each biomarker name to a short array of 1-3 succinct bullet strings that list the textual cues used to decide the value (e.g., ["ER: 'positive reaction 98% 3+' found", "Percent >=1% => Positive"]).

Reference rules for categorization (for model only; do not print these as part of rationale):

- ER/PR: Negative <1%, Positive ≥1%

- Ki-67: Low <10%, Borderline 10–20%, High >20%

- HER2: Negative 0–1+, Equivocal 2+, Positive 3+ (equivocal often reflexed to FISH)

If a field is not present in the report, set it to "Not Reported" and in rationale write a single bullet like "Not found".

Output format (strict JSON). Example:

{{

"tumor_size": "6.0 x 4.0 x 3.5 cm (left)",

"her2": "Ordered, result pending",

"er": "Not Reported",

"pr": "Not Reported",

"ki67": "Not Reported",

"p53": "Not Reported",

"extraction_rationale": {{

"tumor_size": ["Found 'forming a 6.0 x 4.0 x 3.5 cm tumor'"],

"her2": ["Phrase 'Her-2/NEU has been ordered' -> Ordered, result pending"],

"er": ["Not found"]

}}

}}

**EXAMPLE REPORTS FOR CONTEXT:**

Sample Report Extract 1:

*Breast, left, simple mastectomy: Invasive ductal carcinoma, Nottingham grade III (of III), is identified forming a 6.0 x 4.0 x 3.5 cm tumor (AJCC pT3) in the central-upper inner region of the breast. Her-2/NEU has been ordered on paraffin-embedded tissue on the left breast.*

Expected output:

{{

"tumor_size": "6.0 x 4.0 x 3.5 cm (left)",

"her2": "Ordered, result pending",

"er": "Not Reported",

"pr": "Not Reported",

"ki67": "Not Reported",

"p53": "Not Reported"

}}

Report 2: *IHC-stainings:Reported Tumor Size - 1.0 cm left; Estrogen receptor - positive reaction 98% 3+; Progesterone receptor - positive reaction 95% 3+;* *Her-2/neu - negative reaction; Ki67 - 12%.*

Expected output:

{{

"tumor_size": "1.0 cm (left)",

"her2": "Negative (0–1+)",

"er": "Positive (98%, 3+)",

"pr": "Positive (95%, 3+)",

"ki67": "12% (Borderline)",

"p53": "Not Reported"

}}

**Supplementary Table 1
Performance of LLM Models with Its prompting Techniques for Oncology Feature Extraction**

| **Model** | **Prompting** | **Target** | **Accuracy (%)** | **Precision (%)** | **Recall (%)** | **F1 (%)** | **Specificity (%)** |
| --- | --- | --- | --- | --- | --- | --- | --- |
| **GPT-4o-mini** | Zero-shot | HER2 | 79.88 | 56.40 | 46.86 | 51.19 | 89.04 |
|  |  | ER | 94.12 | 92.34 | 92.35 | 92.34 | 96.83 |
|  |  | PR | 93.50 | 92.56 | 93.05 | 92.79 | 96.76 |
|  |  | Tumor Size | 80.80 | 73.92 | 73.92 | 73.74 | 92.95 |
|  | Few-shot (1-shot) | HER2 | 79.91 | 56.93 | 46.08 | 50.92 | 87.90 |
|  |  | ER | 93.30 | 91.92 | 91.04 | 91.46 | 96.27 |
|  |  | PR | 92.56 | 91.90 | 91.59 | 91.74 | 96.10 |
|  |  | Tumor Size | 81.10 | 73.99 | 74.57 | 74.09 | 93.20 |
|  | CoT | HER2 | 95.53 | 59.57 | 54.29 | 56.58 | 88.71 |
|  |  | ER | 94.89 | 92.73 | 93.95 | 93.31 | 97.29 |
|  |  | PR | 93.29 | 92.33 | 92.08 | 92.20 | 96.59 |
|  |  | Tumor Size | 73.16 | 68.92 | 69.60 | 68.49 | 90.97 |
| **GPT-4.1-nano** | Zero-shot | HER2 | 75.72 | 51.79 | 44.90 | 47.94 | 87.44 |
|  |  | ER | 87.86 | 87.46 | 88.78 | 87.71 | 93.19 |
|  |  | PR | 87.86 | 87.03 | 88.09 | 87.35 | 93.94 |
|  |  | Tumor Size | 82.43 | 75.11 | 77.48 | 76.08 | 93.73 |
|  | Few-shot | HER2 | 75.72 | 51.79 | 44.90 | 47.94 | 87.44 |
|  |  | ER | 87.86 | 87.46 | 88.78 | 87.71 | 93.19 |
|  |  | PR | 87.86 | 87.03 | 88.09 | 87.35 | 93.94 |
|  |  | Tumor Size | 82.43 | 75.11 | 77.48 | 76.08 | 93.73 |
|  | CoT | HER2 | 94.22 | 88.07 | 71.91 | 77.34 | 71.91 |
|  |  | ER | 91.79 | 91.25 | 91.86 | 91.32 | 95.41 |
|  |  | PR | 87.54 | 88.20 | 84.98 | 86.21 | 93.13 |
|  |  | Tumor Size | 76.90 | 71.26 | 71.94 | 70.95 | 92.10 |
| **GPT-5-nano** | Zero-shot | HER2 | 88.73 | 58.35 | 41.59 | 46.14 | 77.78 |
|  |  | ER | 69.52 | 82.76 | 62.20 | 65.06 | 81.55 |
|  |  | PR | 68.73 | 80.99 | 61.88 | 64.35 | 81.27 |
|  |  | Tumor Size | 48.25 | 68.96 | 51.30 | 50.66 | 84.48 |
|  | Few-shot | HER2 | 88.73 | 58.35 | 41.59 | 46.14 | 77.78 |
|  |  | ER | 69.52 | 82.76 | 62.20 | 65.06 | 81.55 |
|  |  | PR | 68.73 | 80.99 | 61.88 | 64.35 | 81.27 |
|  |  | Tumor Size | 48.25 | 68.96 | 51.30 | 50.66 | 84.48 |
|  | CoT | HER2 | 94.85 | 86.47 | 77.01 | 80.87 | 77.01 |
|  |  | ER | 94.85 | 93.96 | 92.59 | 93.24 | 97.12 |
|  |  | PR | 93.68 | 93.44 | 92.02 | 92.64 | 96.69 |
|  |  | Tumor Size | 83.37 | 75.41 | 77.31 | 76.28 | 94.13 |
| **Gemini-2.5-flash-lite** | Zero-shot | HER2 | 86.90 | 59.68 | 50.06 | 54.32 | 90.17 |
|  |  | ER | 93.29 | 91.41 | 92.03 | 91.70 | 96.34 |
|  |  | PR | 92.65 | 91.78 | 91.98 | 91.87 | 96.30 |
|  |  | Tumor Size | 81.79 | 74.73 | 75.79 | 75.05 | 93.37 |
|  | Few-shot | HER2 | 87.54 | 54.67 | 39.56 | 43.56 | 77.39 |
|  |  | ER | 90.73 | 89.80 | 88.95 | 89.29 | 94.73 |
|  |  | PR | 89.62 | 89.90 | 87.86 | 88.75 | 94.32 |
|  |  | Tumor Size | 81.47 | 74.36 | 75.64 | 74.89 | 93.29 |
|  | CoT | HER2 | 95.85 | 91.14 | 81.62 | 85.61 | 81.62 |
|  |  | ER | 94.89 | 93.21 | 93.20 | 93.20 | 97.22 |
|  |  | PR | 93.29 | 92.97 | 91.62 | 92.19 | 96.53 |
|  |  | Tumor Size | 81.47 | 73.92 | 76.06 | 74.91 | 93.30 |

**Complete confusion matrices for all four features using all combinations of models and prompting strategies have been given in Supplementary Figures S1-S11. The entire evaluation script along with data can be accessed from our publicly available repository.**

**Figure S1-S11**

**Supplementary figure S12: Confusion matrix comparison of GPT-4o-mini (Chain-of-Thought) and GPT-5-nano (Zero-shot) for triple-negative breast cancer (TNBC) classification.**

**Supplementary Table 2: Summary of clinicopathological characteristics of the selected TCGA-BRCA pathology reports (n = 532)**

| **Characteristic** | **Count (n = 532)** | **Percentage (%)** |
| --- | --- | --- |
| Triple-negative breast cancer (TNBC) cases | 53 | 10.0 |
| Tumor size reported | 468 | 88.0 |
| Tumor size not reported | 64 | 12.0 |
| ER Positive | 220 | 41.4 |
| ER Negative | 68 | 12.8 |
| ER Not Reported | 244 | 45.9 |
| PR Positive | 180 | 33.8 |
| PR Negative | 108 | 20.1 |
| PR Not Reported | 244 | 45.9 |
| HER2 Positive | 50 | 9.4 |
| HER2 Negative | 228 | 42.9 |
| HER2 Not Reported | 254 | 47.7 |
